# Hrs- and CD63-Dependent Competing Mechanisms Make Different Sized Endosomal Intraluminal Vesicles

**DOI:** 10.1111/tra.12139

**Published:** 2014-01-08

**Authors:** James R Edgar, Emily R Eden, Clare E Futter

**Affiliations:** 1 UCL Institute of Ophthalmology11-43 Bath Street, London, EC1V 9EL, UK

**Keywords:** CD63, cholesterol, Hrs, intraluminal vesicle, multivesicular endosome

## Abstract

Multivesicular endosomes/bodies (MVBs) contain intraluminal vesicles (ILVs) that bud away from the cytoplasm. Multiple mechanisms of ILV formation have been identified, but the relationship between different populations of ILVs and MVBs remains unclear. Here, we show in HeLa cells that different ILV subpopulations can be distinguished by size. EGF stimulation promotes the formation of large ESCRT-dependent ILVs, whereas depletion of the ESCRT-0 component, Hrs, promotes the formation of a uniformly sized population of small ILVs, the formation of which requires CD63. CD63 has previously been implicated in ESCRT-independent sorting of PMEL in MVBs and transfected PMEL is present on the small ILVs that form on Hrs depletion. Upregulation of CD63-dependent ILV formation by Hrs depletion indicates that Hrs and CD63 regulate competing machineries required for the generation of distinct ILV subpopulations. Taken together our results indicate that ILV size is influenced by their cargo and mechanism of formation and suggest a competitive relationship between ESCRT-dependent and -independent mechanisms of ILV formation within single MVBs.

Multivesicular endosomes/bodies (MVBs) are endosomal compartments, so called because of their content of intraluminal vesicles (ILVs). The first function of MVBs to be identified was the sorting of proteins, like activated EGF receptor (EGFR), to the lysosome. Lysosomally directed membrane proteins are sorted onto the ILVs of MVBs, whereas recycling proteins are retained on the perimeter membrane from where they are returned to the cell surface. When all the recycling proteins are removed, the mature MVB delivers its contents to lysosomal compartments through full fusion or transient ‘kiss-and-run’ events (1–3). More recently it has become clear that MVBs can have alternative fates, including fusion with the cell surface to release the ILVs as exosomes 4, and in melanogenic cells MVBs are intermediates on the pathway leading to melanosome biogenesis 5. Whether MVBs with different destinations exist as entirely separate entities is unclear. We and others have shown that there are multiple populations of MVBs which differ in their protein and lipid composition (6,7). Furthermore, more than one mechanism of making ILVs and sorting cargo onto them have been described (8,9). The extent to which different mechanisms of ILV formation and sorting operate within the same MVB is unclear.

The importance of MVBs in the regulation of multiple cellular functions, including receptor tyrosine kinase signalling, the biogenesis of lysosome-related organelles and intercellular communication has led to great interest in the mechanisms regulating ILV generation and the selective inclusion of cargo on them. The best characterized MVB sorting mechanism is the ESCRT machinery, which is composed of four protein complexes (ESCRTs-0, -I, -II, -III). Ubiquitinated cargos are first recognized and bound by the ESCRT-0 complex, and subsequently passed to later ESCRT components, which also mediate ILV formation (10,11). ESCRT-0, -I and -II contain ubiquitin-binding domains which allow the passage of cargos between ESCRT complexes on the endosomal membrane. In mammalian cells, depletion of components of all four ESCRT complexes does not abolish MVB formation 12, and a number of ESCRT-independent mechanisms of ILV formation have been reported. In an oligodendroglial cell line, the sphingolipid ceramide is required for the formation of ILVs that are subsequently released as exosomes 13. In melanogenic cells, PMEL is targeted to ILVs in an ESCRT- and ubiquitin-independent manner 14. Within MVBs, PMEL undergoes limited proteolysis whereupon it induces the formation of striations upon which melanin is deposited 15. Targeting of the luminal portion of PMEL to ILVs requires the tetraspanin CD63, but, interestingly, the cytoplasmic portion of PMEL that remains after proteolysis is targeted to lysosomes in an ESCRT-dependent manner, implying that ESCRT-dependent and -independent mechanisms can operate on contiguous membranes 16.

ESCRT-independent mechanisms are likely to exist in all cells but the level to which different mechanisms predominate may depend on the cell type and the function/fate of the MVBs. In addition, as yet uncharacterised ESCRT-independent mechanisms of ILV formation may exist. In this study, we have taken HeLa cells, where ESCRT-dependent ILV formation has been well characterized, to determine whether different mechanisms of ILV formation operate within single MVBs. We show that different populations of ILVs can form within single MVBs that can be distinguished on the basis of size and mechanism of formation. CD63-dependent formation of small ILVs is partially suppressed in HeLa cells by Hrs-dependent formation of larger ILVs.

## Results

### Neither CD63 nor ceramide depletion inhibit ILV formation in resting HeLa cells

**Figure 1:**
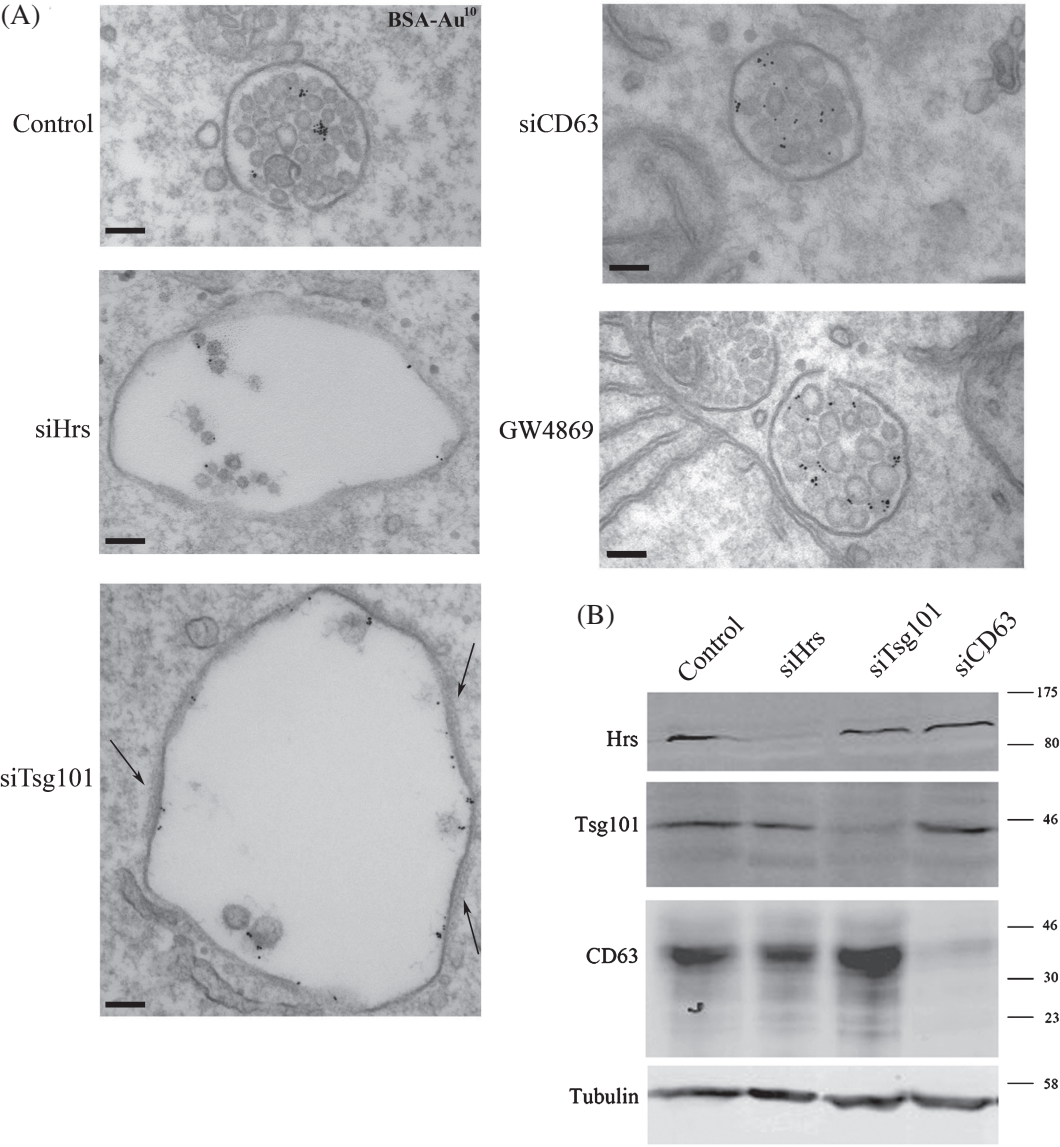
Depletion of Hrs and Tsg101 but not CD63 or ceramide affects the morphology of MVBs in resting HeLa cells. HeLa cells were treated with non-targeting control, Hrs, Tsg101 or CD63 siRNA or were treated with the sphingomyelinase inhibitor, GW4869, to deplete ceramide. Cells were then incubated for 2 h in the presence of BSA-gold and prepared for conventional EM. Clathrin coats in Tsg101-depleted cells are shown by arrows (A). Knockdown efficiency was analysed by western blotting (B). Scale bar: 100 nm.

HeLa cells were depleted of either the ESCRT-0 protein Hrs, ESCRT-I protein Tsg101 or the tetraspanin CD63, or were treated with GW4869 to inhibit sphingomyelinase and thereby deplete cellular ceramide. The cells were then incubated with BSA-gold for 2 h prior to fixation to aid identification of endosomal compartments and MVBs were identified as vacuoles containing one or more ILVs and BSA-gold (Figure 1A). MVBs could be distinguished from lysosomes because lysosomes also contain irregular membrane whorls 17. Knockdown efficiencies were estimated at 86.5% for Hrs, 81.6% for Tsg101 and 84.1% for CD63 (Figure 1B).

**Figure 2:**
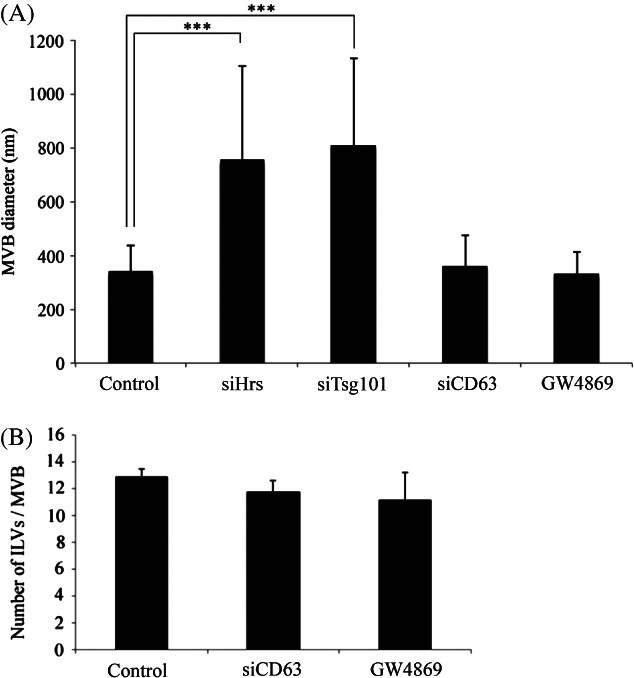
CD63 or ceramide depletion does not affect MVB diameter or ILV number. HeLa cells were treated with non-targeting control, Hrs, Tsg101 or CD63 siRNA or were treated with the sphingomyelinase inhibitor, GW4869, to deplete ceramide. Cells were then incubated for 2 h with BSA-gold and analysed by conventional EM. Diameters and ILV numbers of BSA-gold-containing MVBs were quantified in random sections. Hrs or Tsg101 depletion caused enlargement of MVBs (***p < 0.001), whilst CD63 or ceramide depletion had no effect on MVB size compared to control cells (A). CD63 or ceramide depletion had no significant effect on the number of ILVs per MVB (B). ***p < 0.001. Data shown are mean ± SD (50–100 MVBs/condition, 3 independent experiments).

As shown in Figure 1 and consistent with previous observations (18,19), Hrs and Tsg101 depletion induced the generation of enlarged MVBs many of which contained very few ILVs. In contrast, depletion of the ESCRT-independent candidates, CD63 or ceramide, caused no clear morphological change. Quantitative analysis confirmed that CD63 or ceramide depletion had no effect on MVB diameter or ILV number (Figure 2A,B).

Analysis of the number of ILVs per MVB in Hrs- or Tsg101-depleted cells is complicated because of the dramatically increased MVB diameter. Some of the Hrs-depleted MVBs contained 50+ ILVs, whereas others contained very few and we predict that with the enlarged diameters and increased luminal volume, ILVs may not be homogeneously distributed. Therefore, quantitation of ILV numbers in Hrs- or Tsg101-depleted cells was not performed.

Thus, in resting HeLa cells ILV formation appears to be inhibited by depletion of ESCRT components, but is not inhibited by depletion of components (ceramide and CD63) which have previously been implicated in ESCRT-independent MVB sorting. Nevertheless, after depletion of Hrs or Tsg101, some ILVs can form, probably in an ESCRT-independent manner.

### EGF stimulation promotes the formation of large ILVs in EGFR-containing MVBs

**Figure 3:**
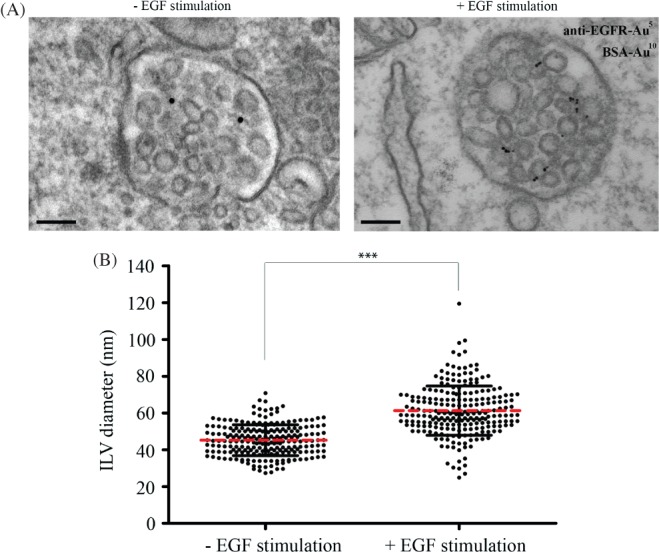
EGF stimulation promotes the generation of large ILVs. HeLa cells were incubated with 10 nm BSA-gold for 2 h at 37°C and stimulated with or without EGF and 5 nm anti-EGFR gold for the final 25 min prior to fixation. Cells were analysed by conventional EM (A) and ILV sizes in BSA-gold-containing MVBs measured (B). Data shown are the mean ± SD (>200 ILVs/condition, 3 independent experiments). Scale bar: 100 nm.

**Figure 4:**
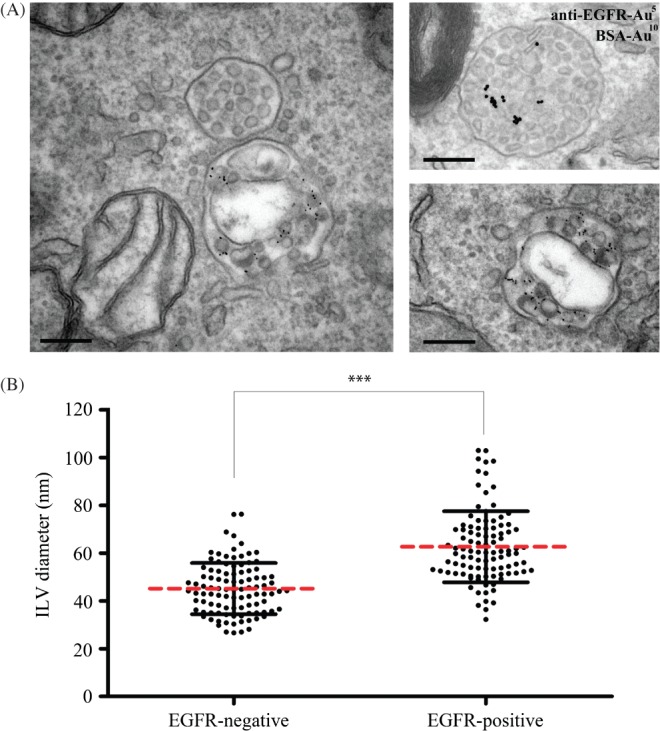
Large ILVs are only found in MVBs that contain EGFR. HeLa cells were incubated with 10 nm BSA-gold for 2 h at 37°C and stimulated with or without EGF and 5 nm anti-EGFR gold for the final 25 min prior to fixation. Cells were analysed by conventional EM (A) and the sizes of 100 ILVs in EGFR-gold positive and -negative MVBs were measured (B). Scale Bar: 200nm.

Depletion studies have the potential limitation of incomplete knockdown, which could lead to incomplete inhibition of ILV formation. Furthermore, our knowledge of ESCRT-independent mechanisms of ILV formation is likely to be incomplete, limiting our ability to inhibit ESCRT-independent ILV formation. An alternative approach to determine whether there are multiple mechanisms of ILV formation in the same MVB is to analyse the ILVs themselves and determine whether multiple populations can be distinguished either on the basis of size or cargo. We therefore measured the sizes of ILVs in resting cells and following EGF treatment, which we had previously shown to upregulate ESCRT-dependent ILV formation 6. As shown in Figure 3A, a range of ILV sizes could be observed in the absence and the presence of EGF stimulation, but there was a greater range of ILV sizes after EGF stimulation. The section plane could explain some variation in ILV size but quantitative analysis showed that EGF stimulation caused an increase in mean ILV size and EGF stimulation induced the formation of a population of ILVs that were larger than any observed in the absence of EGF stimulation (Figure 3B). In porcine aortic endothelial cells stably expressing wild-type, but not ubiquitination-deficient, EGFR 20, EGF stimulation also induced the formation of ‘large’ ILVs (not shown). By co-incubating the cells with an anti-EGFR antibody conjugated to colloidal gold it was possible to identify MVBs that did and did not contain EGFR. In EGF-stimulated cells, non-EGFR-containing MVBs showed ILV sizes similar to those in unstimulated cells whereas MVBs that contained EGFR showed increased mean ILV size and the very large subpopulation of ILVs (Figure 4). We have previously shown that EGF stimulation upregulates MVB formation, as well as ILV formation 6, although EGFR can also enter pre-existing MVBs following EGF stimulation. To determine whether the large ILVs are present only in the subset of MVBs induced by EGF stimulation, ILVs were subdivided into ‘small’ (<40 nm) and ‘large’ (>40 nm). In resting cells, most MVBs contained both small and large ILVs but a proportion contained exclusively small or large. In EGF-stimulated cells, the MVB population containing exclusively small ILVs was absent, indicating that EGF stimulation promotes the formation of enlarged ILVs in MVBs whose presence does not depend on EGF stimulation, as well as in MVBs formed as a result of EGF stimulation (Figure S1A, Supporting Information). Importantly, MVBs containing both small and large ILVs are present in both resting and EGF-stimulated cells (Figure S1B).

Although ESCRT-dependent and -independent mechanisms of ILV formation probably coexist in HeLa cells, EGF stimulation specifically upregulates ESCRT-dependent ILV formation. Therefore, one explanation for increased mean ILV size following EGF stimulation could be that ESCRT-dependent ILVs are larger than those formed ESCRT-independently. However, the demonstration that EGF stimulation results in the formation of ILVs that are larger than any formed in non-stimulated cells suggests that EGF stimulation itself, either through signalling or through cargo (EGFR), might cause increased ILV size.

### Hrs depletion but not Tsg101 depletion prevents the EGF-dependent formation of enlarged ILVs but promotes the formation of smaller ILVs than in resting cells

**Figure 5:**
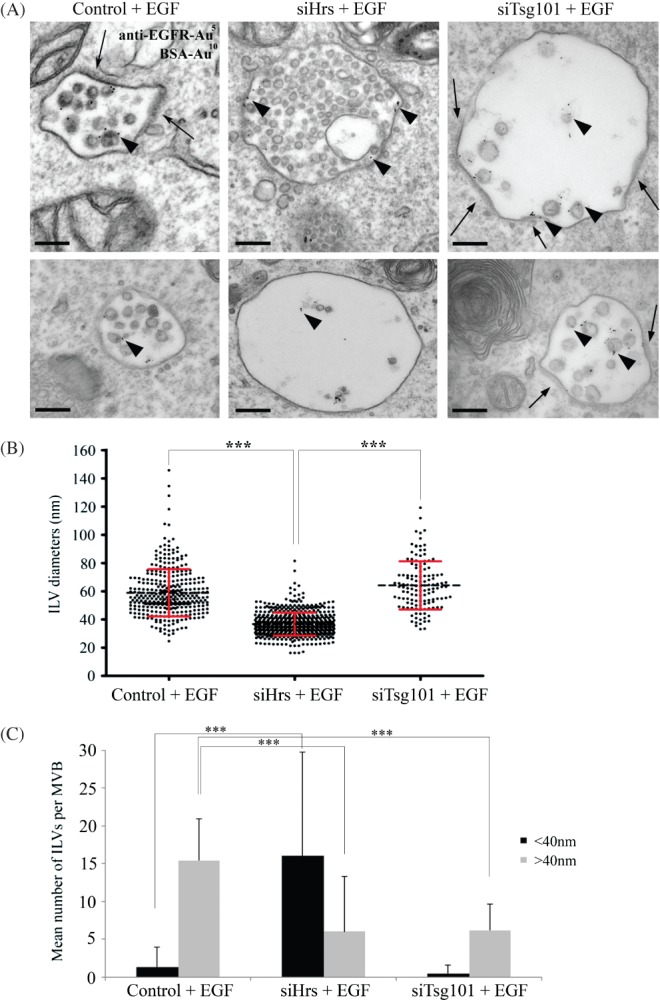
Hrs-depleted HeLa cells do not display enlarged ILVs following EGF stimulation. Control, Hrs- or Tsg101-depleted cells were incubated with 10 nm BSA-gold for 2 h at 37°C and stimulated with EGF and 5 nm anti-EGFR-gold for 25 min prior to fixation. Cells were analysed by conventional EM (A). Arrowheads indicate EGFR-gold. Endosomal clathrin coats are indicated by arrows. The diameters of all ILVs within 20 MVBs were analysed (B). Binning the ILV diameters to either <40 or >40 nm (C) reveals a greatly increased number of smaller ILVs/MVB in Hrs-depleted cells. Data are mean ± SD (2 independent experiments). ***p < 0.001. Scale bar: 200 nm

If the large ILVs formed following EGF stimulation are ESCRT-dependent then their formation should be inhibited by depletion of components of the ESCRT machinery. HeLa cells were therefore depleted of Hrs or Tsg101 and then incubated with EGF and anti-EGFR gold for 25 min. As with unstimulated cells (Figure 1) Hrs and Tsg101-depletion in EGF-stimulated cells induced the formation of enlarged MVBs that frequently contained very few ILVs (Figure 5A) in agreement with previous studies (18,19). Equal numbers of anti-EGFR-gold positive MVBs were analysed from control, Hrs-depleted or Tsg101-depleted HeLa cells and the diameter of every ILV was measured. In Hrs-depleted cells, the EGF-dependent formation of large ILVs was prevented and, surprisingly, the ILVs that did form were uniformly very small (Figure 5B). In contrast, in Tsg101-depleted cells, although the total number of ILVs/MVB was reduced, the mean size of the ILVs that did form was the same as that in control cells. In control EGF-stimulated cells the majority of ILVs had a diameter of greater than 40 nm. The formation of these ‘large’ ILVs was reduced to similar extents by Hrs and Tsg101 depletion (Figure 5C). However, Hrs-depleted cells contained many ILVs with a diameter of less than 40 nm. While we cannot exclude the possibility that some small ILVs were obscured by the presence of the large ILVs in control cells, their number was clearly increased following Hrs depletion. Depletion of Hrs, but not Tsg101, also induced the formation of ‘small’ ILVs in EGF-stimulated H4 neurogliomal cells (not shown).

On EGFR-containing MVBs electron-dense coats could frequently be observed on flattened domains of the perimeter membrane of endosomes (Figure 5A – arrows). These coats have previously been shown to contain clathrin that is recruited by Hrs (21,22). As expected, the formation of these coats was inhibited by Hrs depletion. In contrast, in Tsg101-depleted cells the electron-dense coats were expanded in both EGF-stimulated (Figure 5A) and resting (Figure 1A) cells and frequently extended over large areas of the perimeter membrane of enlarged MVBs, indicating that in the absence of Tsg101 their disassembly is inhibited.

**Figure 6:**
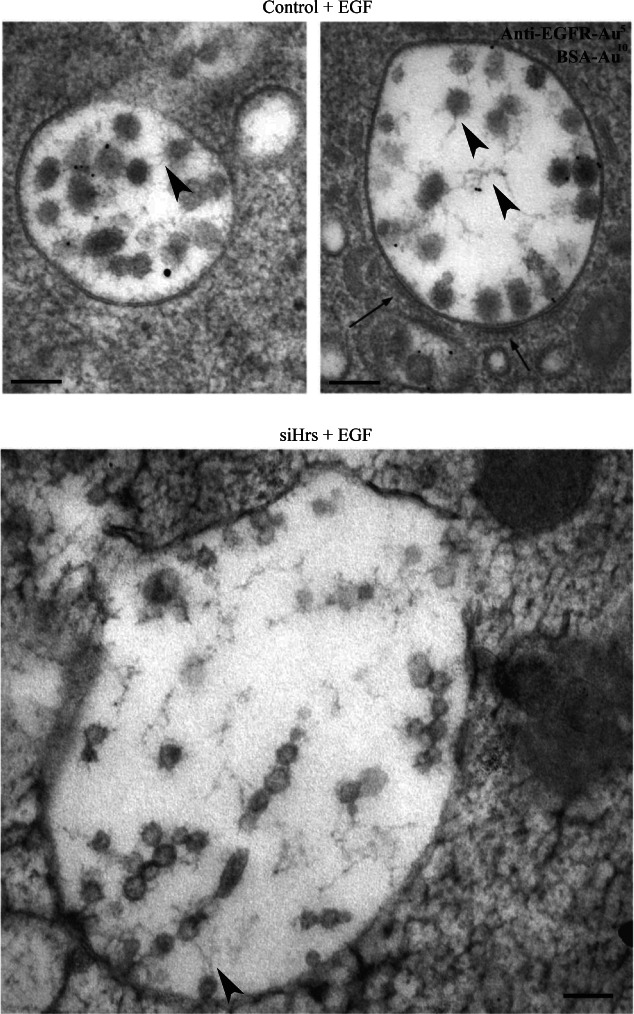
High-pressure freezing reveals that ILVs may be connected by intramembranous material. Control or Hrs-depleted HeLa cells were incubated with 10 nm BSA-gold for 2 h at 37°C. Cells were stimulated with EGF and 5 nm anti-EGFR-gold for 25 min prior to fixation and then analysed by conventional EM. Endosomal clathrin coats are shown by arrows. Intramembranous fibrils are shown by arrowheads. Scale bar: 100 nm

The small ILVs formed in Hrs-depleted cells were only present in a subset of MVBs. This could arise because, in chemically fixed preparations the small ILVs are not evenly distributed within the lumen of the enlarged MVBs that form on Hrs depletion, leading to their presence depending on the section plane through the MVB. Furthermore, chemical fixation can cause shrinkage of cells and tissue. Therefore, the effects of Hrs depletion and EGF stimulation on ILV formation were examined in high-pressure frozen (HPF) cells (Figure 6). Hrs depletion induced the formation of small ILVs of similar sizes to those observed in chemically fixed preparations (Figure S2) and the lipid bilayer surrounding the small ILVs could be more readily observed in these HPF cells (Figure 6). Furthermore, the spatial localisation of ILVs within the enlarged MVBs following Hrs depletion is better preserved in HPF cells, such that, unlike chemically fixed preparation where MVBs were either densely packed with small ILVs or were devoid of them, most MVBs contained small ILVs. The small ILVs were frequently connected by small fibres which sometimes connected long strings of ILVs (Figure 6).

### The formation of small ILVs in Hrs-depleted cells is not prevented by incubation in delipidated serum

**Figure 7:**
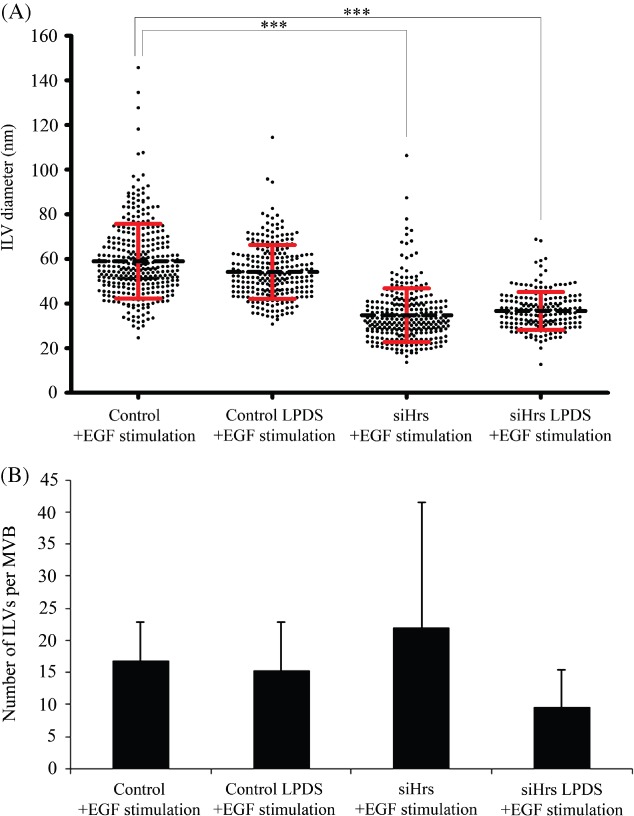
Small ILVs still form in Hrs-depleted cells cultured in delipidated serum. Control or Hrs-depleted cells were cultured in the presence of LPDS prior to EGF stimulation for 25 min and prepared for conventional EM. Diameters of all ILVs within 20 MVBs were analysed/specimen. The ILVs of Hrs-depleted LPDS-treated cells had the same mean diameter as those cultured in full serum (A) but had a reduced number of ILVs per MVB (B). Data shown are the mean ± SD (approximately 20 MVBs/condition).

One recently described role of Hrs that is independent of the ESCRT machinery is regulation of endosomal cholesterol levels. Hrs, but not Tsg101 depletion, has recently been shown to cause low-density lipoprotein (LDL)-derived cholesterol accumulation in endosomes 23. In keeping with the results of Du et al., we found that Hrs depletion-induced cholesterol accumulation in endosomes that could be prevented by culturing the cells with medium containing lipoprotein-deficient serum (LPDS) (Figure S3). The presence of a lipid bilayer surrounding the small ILVs formed on Hrs depletion (Figure 6) demonstrated that they were not LDL particles. The possibility remained that cholesterol accumulation on the perimeter membrane of MVBs in Hrs-depleted cells could lead to membrane instability and spontaneous inward budding to form the small ILVs. However, culture of EGF-stimulated control or Hrs-depleted cells in LPDS had no effect on mean ILV size, indicating that small ILV production in Hrs-depleted cells is not dependent on LDL-derived cholesterol (Figure 7). However, there was a reduction in the mean number of ILVs/MVB in Hrs-depleted but not control cells cultured in LPDS, suggesting that cholesterol may promote the formation of the small ILVs (Figure 7B). It should, however, be noted that in the presence or absence of LPDS, Hrs-depleted MVBs contained a wide range in the number of ILVs.

### Small ILVs formed following Hrs depletion carry CD63 and the CD63 cargo, PMEL

**Figure 8:**
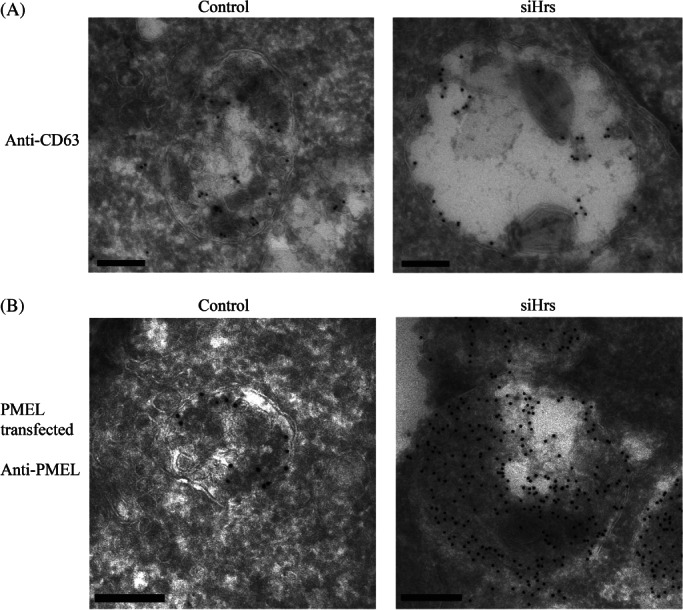
CD63 and PMEL are cargos for small ILVs generated following Hrs depletion. Control or Hrs-depleted cells were prepared for cryo-immuno EM and ultrathin frozen sections labelled with anti-CD63 antibody (A). CD63 was present on ILVs in control cells and on small ILVs in Hrs-depleted cells. Control or HeLa cells depleted of Hrs were transfected with PMEL (B). Cryo-immuno EM of these cells with anti-PMEL antibody revealed that PMEL was present on small ILVs in Hrs-depleted cells but the tight packing of ILVs and PMEL-induced fibrils in control and Hrs-depleted cells prevented the determination of whether it was exclusively on small ILVs. Scale bar: 200 nm

Why are the ILVs formed on Hrs depletion small? Is it because they are formed ESCRT-independently by a mechanism that makes smaller ILVs than those formed in an ESCRT-dependent manner? An alternative possibility is that these ILVs are ESCRT-dependent but have reduced amounts of protein cargo, rendering them smaller. We therefore performed cryo-immuno electron microscopy (EM) to search for possible protein cargos for the small ILVs formed on Hrs depletion. In Hrs-depleted cells, the small ILVs tend to fall out of cryosections, particularly in the absence of glutaraldehyde fixation. This may in part explain why they have not been reported before. With the addition of 0.1% glutaraldehyde some small ILVs were retained and these stained for CD63, indicating that they do indeed contain protein cargo (Figure 8A). In control cells, CD63 was clearly present on ILVs but the dense packing of ILVs within MVBs, together with the potential distance of a gold particle from the antigen (up to ∼25 nm), made it difficult to tell whether CD63 localized exclusively to small ILVs (Figure 8A).

CD63 has previously been shown to be required for the ESCRT-independent sorting of the luminal domain of PMEL to ILVs 16. To determine whether PMEL was targeted to the subpopulation of small ILVs formed following Hrs depletion, HeLa cells were transfected with PMEL in the presence and absence of Hrs depletion. When expressed in HeLa cells PMEL has previously been shown to be targeted to ILVs and induce the formation of fibrils within MVBs 15. Despite the dense packing of ILVs in control cells, together with the presence of fibrils in both control and Hrs-depleted cells, it was possible to discern PMEL staining on the small ILVs in control and Hrs-depleted cells and, in agreement with previous findings, depletion of Hrs did not prevent the targeting of PMEL to ILVs (Figure 8B). It was not possible from these data to establish that CD63 or PMEL localized exclusively to the small subpopulation of ILVs but they do indicate that the small ILVs can traffic these two cargos.

### Production of small ILVs following Hrs depletion is inhibited by CD63 depletion

**Figure 9:**
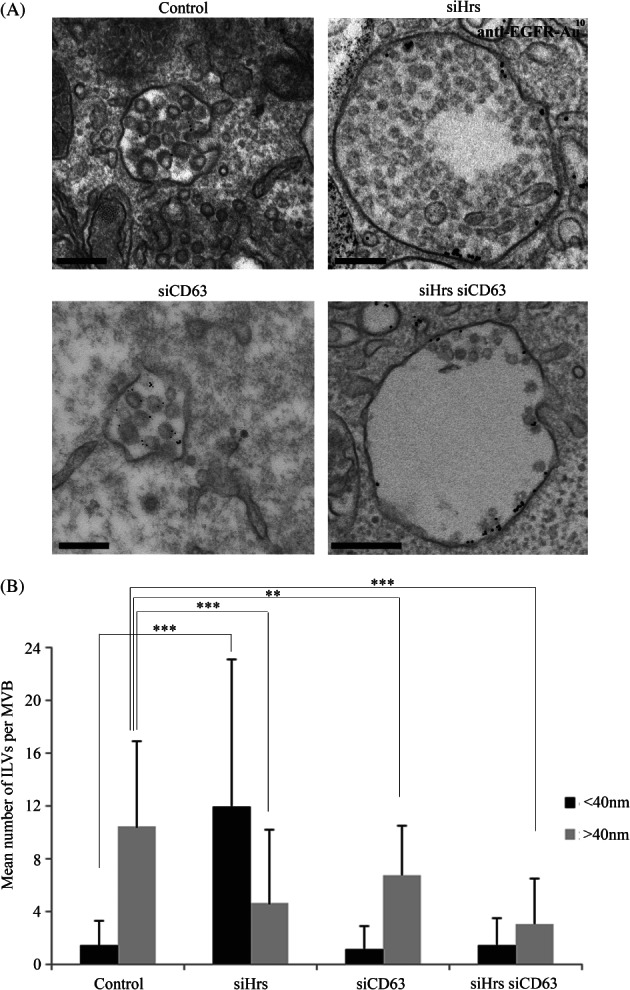
Simultaneous depletion of Hrs and CD63 prevents the formation of small ILVs. HeLa cells were depleted of Hrs or CD63 alone or Hrs and CD63 and were incubated in the presence of EGF and anti-EGFR gold for 25 min prior to fixation. Cells were analysed by conventional EM (A). Binning the ILV diameters to either <40 nm or >40 nm revealed that simultaneous CD63 depletion prevented the increase in number of smaller ILVs/MVB induced by Hrs depletion (B). Data shown are the mean ± SD (>40 MVBs/condition, 2 independent experiments). Scale bar: 200 nm.

CD63 has previously been implicated in ESCRT-independent sorting within MVBs 16. The demonstration that the small ILVs formed on Hrs depletion were positive for CD63 raised the possibility that they might be formed by an ESCRT-independent mechanism involving CD63. HeLa cells were depleted of Hrs or CD63 alone or concomitantly depleted of Hrs and CD63, and then EGF stimulated in the presence of anti-EGFR-gold for 25 min. As previously shown, Hrs depletion inhibited the formation of large ILVs (>40 nm) and induced the formation of small ILVs (<40 nm) (Figure 9). CD63 depletion partially inhibited the formation of large ILVs in EGF-stimulated cells (Figure 9) without affecting their formation in resting cells (Figure S4). CD63 depletion almost completely prevented the formation of the small ILVs (under 40 nm) induced by Hrs depletion in both EGF-stimulated and resting cells (Figures 9 and S4). That we still observe some ILVs in cells depleted of both Hrs and CD63 raises the possibility of further mechanisms of ILV production.

## Discussion

Our results agree with previous findings that while EGF-stimulated ILV formation depends on the ESCRT machinery, ESCRT-independent mechanisms of ILV generation also exist in HeLa cells 12. Our data further suggest that ESCRT-dependent and -independent ILV formation can operate within the same MVBs.

We have shown that ILVs of different sizes coexist within MVBs, and that variation in ILV size increases upon EGF stimulation. Apparent variation in ILV size in random sections is not simply due to varying plane of section through the ILVs because EGF stimulation increases mean ILV size. Could it be that nature and amount of cargo influences ILV size or are ILVs of different sizes formed by different mechanisms? Our data provides evidence for both possibilities. EGF stimulation upregulates ESCRT-dependent ILV formation (12,18) and so the increase in mean ILV size under these conditions could be because ESCRT-dependent ILVs are larger than ESCRT-independent ILVs. However, although ESCRT-dependent ILVs do form in the absence of EGF stimulation, some of the ILVs formed after EGF stimulation are larger than any seen in resting cells indicating some effect of either EGF signalling or cargo. Components of the ESCRT machinery, including Hrs, become phosphorylated in response to EGF stimulation 24. How this phosphorylation affects ESCRT activity is not clear but it is conceivable that ESCRT phosphorylation might modulate ILV size. Perhaps more likely is that increased ubiquitinated cargo (EGFR) leads to larger ILVs. The presence of ubiquitinated cargo has previously been shown to stabilize ESCRT components on the endosomal membrane and to promote ILV formation 25. Our data suggests that the amount of ubiquitinated cargo may also increase ILV size. The size of ESCRT-dependent ILVs could depend on the size of patches of ubiquitinated cargo on the perimeter membrane of the MVB that then invaginate to form the ILV. Consistent with the idea that the size of ESCRT assemblies on the MVB perimeter influences ILV size is the demonstration that regulators of the ATPase responsible for ESCRT disassembly modulate ILV size 26.

Hrs depletion caused a reduction in mean ILV size such that ILVs in Hrs-depleted cells were smaller than those in resting cells. Unlike the ILVs seen in control cells following EGF stimulation, which were quite variable in diameter, the ILVs in Hrs-depleted cells were uniformly very small. The fact that these very small ILVs were not induced by Tsg101 depletion raised the possibility that the small ILVs might be caused by loss of an Hrs function that is independent of the rest of the ESCRT machinery. Hrs depletion has recently been shown to cause accumulation of LDL-derived cholesterol in the endocytic pathway 23 in a manner independent of components of ESCRT-I, -II and -III. Increased cholesterol content could induce small ILV formation through effects on membrane fluidity, promoting membrane curvature 27, or could act as a platform where factors involved in budding accumulate. Alternatively, Hrs depletion may alter the membrane organization, as a recent study has shown that *in vitro* ESCRT proteins induce lipid phase separation and that this may aid membrane budding 28. However, our demonstration that there was no change in mean size of ILVs in the absence of LDL-derived cholesterol indicates that the small size of the ILVs was not due to cholesterol accumulation.

How else could Hrs and Tsg101 depletion have different effects? Recent elegant studies have investigated the contributions of different components of the ESCRT machinery to ILV formation *in vitro* using purified yeast ESCRT complexes (29,30). While some controversy remains over the roles of ESCRTs-I, -II and -III in membrane deformation, the primary role of ESCRT-0 is in cargo concentration rather than membrane deformation. Is it therefore possible that in the absence of Hrs, ESCRT-dependent budding continues but with little or no ubiquitinated cargo and it is the reduced amount of cargo that leads to the formation of the very small ILVs? However, a recent study in yeast showed that the absence of ubiquitinated cargo suppressed ILV formation rather than generating smaller ILVs 25. Furthermore, although Hrs/ESCRT-0 has been implicated primarily in cargo concentration rather than ILV formation, Hrs does have a role in recruiting ESCRT-I and thereby later components of the ESCRT machinery 31 and so its depletion would be expected to inhibit ESCRT-dependent ILV formation.

The small ILVs do have cargo (CD63 and PMEL when it is expressed), and their formation is suppressed by CD63 depletion, which has been implicated in ESCRT-independent budding events 16. Our data strongly suggests that CD63 plays a role not only in sorting of cargo like PMEL onto ILVs, as has been previously shown 16, but also in ILV formation. CD63 is a tetraspannin protein that can potentially partition into lipid microdomains where it could have a direct role in membrane deformation or could recruit other components of ESCRT-independent budding machineries. Although the possibility that the later ESCRTs (I, II and III) could be involved in the formation of the small ILVs cannot be excluded, an alternative possible explanation for their absence in Tsg101-depleted cells is the presence of large clathrin-coated domains on the MVB perimeter. That these domains, which do not form in Hrs-depleted cells, might be inhibitory to ILV formation has been previously suggested 32 and is consistent with the demonstration that ESCRT-dependent ILVs tend to form at the edges of these domains (22,32). It is likely that these domains would be inhibitory to ESCRT-independent ILV formation as well and so their accumulation may prevent the formation of the small Hrs-independent ILVs that occurs in Hrs-depleted cells. It should be noted, however, that in Tsg101-depleted melanogenic cells ESCRT-independent ILVs containing PMEL still formed 16. It is possible, therefore, that the presence of a CD63-dependent cargo, such as PMEL, can promote the formation of ESCRT-independent ILVs, and/or that in melanogenic cells the CD63-dependent pathway predominates. In HeLa cells, ESCRT-dependent ILV formation appears to predominate because, although some small CD63-dependent ILVs form in non-Hrs-depleted cells, their formation is greatly increased upon Hrs depletion. The balance between Hrs-dependent and CD63-dependent mechanisms may be of critical importance in determining MVB and ILV fate. Embryonic fibroblasts from CD63 knockout mice exhibit no clear defect in endocytic or lysosomal transport 33, suggesting that in these cells, like HeLa cells, the ESCRT-dependent pathway predominates. However, in pigmented retinal pigment epithelial cells of the same CD63 knockout mouse there are defects in melanosome formation 16, suggesting that during melanosome biogenesis the CD63 pathway is active and the ESCRT pathway may be suppressed. That ESCRT-dependent and ESCRT-independent budding can occur within the same MVB is agreement with the results of van Niel et al. 16. They proposed that CD63 co-ordinates ESCRT-dependent and ESCRT-independent budding events on contiguous membranes and protects cargos, like full length PMEL, from ESCRT-dependent ILV sorting and degradation. That CD63 depletion partially inhibited ESCRT-dependent budding in EGF-stimulated but not resting cells is consistent with CD63 playing a role in co-ordination between ESCRT-dependent and-independent budding events. Our data further suggests that the early components of the ESCRT machinery may also play a role in regulating the balance between ESCRT-dependent and -independent ILV formation. That in EGF-stimulated cells ESCRT-dependent ILV formation predominates and in PMEL-expressing cells the CD63-dependent pathway is more active suggests that the relative amounts of ESCRT-dependent and -independent cargo play a role in co-ordination between the two mechanisms. If Hrs-dependent and -independent mechanisms coexist in the same MVB there must be a mechanism for segregating the different ILVs which, at least in melanogenic cells, would have different destinations. One possibility is that ILVs may be spatially connected within the lumen of the MVB. In high-pressure frozen samples, we observed a number of small connections between ILVs that sometimes appeared as ‘threads’ and intermembranous fibrillar material has previously been observed within MVBs 34. One possibility is that these threads may act to spatially segregate separate ILV populations, such that MVBs may deliver contents to multiple compartments through ‘kiss-and-run’ activity.

We have demonstrated that MVBs contain more than one population of ILVs that are formed by different mechanisms, and that a CD63-dependent mechanism can be suppressed by an Hrs-dependent mechanism. The tight packing of ILVs within MVBs makes it difficult to distinguish them on the basis of cargo but our data suggests that ILV size could allow the identification of different subpopulations of MVB and would therefore aid in the analysis of mechanisms that regulate segregation of ILVs and their ultimate fates.

## Materials and Methods

### Cell culture

HeLa cells (ATCC) were cultured in DMEM/10%FCS or 10% LPDS (Millipore) for 18–24 h in 5% CO_2_. Prior to EGF stimulation cells were serum-starved for 90 min and then incubated with EGF (100 ng/mL) and gold probes in DMEM containing 0.2% BSA. To deplete cells of ceramide, cells were cultured in GW489 hydrate (Sigma) at a final concentration of 5 ng/mL for 24 h in full media.

### Transfections

Cells were transfected with small interfering RNA (siRNA) using Oligofectamine (Invitrogen) on days 1 and 2 and harvested on day 4. siRNA duplexes were synthesized by Dharmacon, the target sequences were: 5′-CCA GUC UUC UCU CGU CCU A-3′ (Tsg101), 5′-AGA GAC AAG UGG AGG UAA A-3′ (Hrs), both as previously described 18, 5′-GTT CTT GCT CTA CGT CCT C-3′ (CD63), as previously described 16. Negative control siRNA was used for control cells (Mammalian AllStar negative siRNA, Qiagen). Efficiency of knockdown was assessed by western blotting using anti-Hrs (Enzo Lifesciences), anti-Tsg101 (Genetex) and anti-CD63 (1B5, a generous gift from Mark Marsh, University College, London) antibodies.

For concurrent depletion and expression studies, HeLa cells were transfected with siRNA on days 1 and 2 and transfected with PMEL (a generous gift from Michael Marks, University of Pennsylvania) on day 3 using Lipofectamine 2000 reagent (Invitrogen), following manufacturer's guidelines.

### Conventional electron microscopy

BSA and monoclonal antibody to the extracellular domain of the EGF receptor (108) were coupled to colloidal gold as described 35. Cells were cultured on 13 mm diameter Thermanox**®** coverslips (Nalge Nunc International), and after appropriate treatments were chemically fixed and processed for conventional EM as previously described 36.

For high-pressure freezing, cells were cultured on dishes, washed and scraped in ice-cold PBS, then pelleted. The cell pellet was gently removed into a copper specimen carrier (Leica) and high-pressure frozen in a Leica EM PACT high-pressure freezer. Samples were transferred to a Leica EM AFS2 for freeze substitution in 0.1% tannic acid in acetone (22 h, −90°C), acetone (2 h, −90°C) and then 2% osmium tetroxide (8 h, −90°C, 8 h −60°C). Samples were washed in acetone and gradually heated to room temperature before dehydration in propylene oxide and embedding in Epon.

Sections of 70 nm were stained with lead citrate and examined in a Joel 1010 EM. image J was used to measure the diameters of MVBs and ILVs. MVBs were identified by morphology, having only discrete ILVs and monodisperse gold. Lysosomes contain multilamellar profiles and aggregated gold. The maximum measurable distance for each MVB or ILV was measured. At least 20 MVBs were analysed per experiment from separate cells and data was analysed from duplicate or triplicate separate experiments. Box scatter plots were generated using Prism software and statistical tests were performed in Microsoft Excel. Two-tailed, unpaired *t*-tests were used to analyse the statistical differences between MVB and ILV diameters.

### Immunoelectron microscopy

Cells were fixed with 4% paraformaldehyde (PFA)/0.1 M phosphate buffer pH 7.4 or 4% PFA/0.1% glutaraldehyde/0.1 M phosphate buffer pH 7.4, infused with 2.3 M sucrose and supported in 12% gelatine. Approximately 70 nm sections were cut using a Leica Reichert ultracut S EM-FCS cryo-ultramicrotome at −120°C. The sections were picked up using a 1:1 mixture of methylcellulose and 2.3 M sucrose and dropped onto formvar and carbon-coated hexagonal 100-mesh copper grids (Agar scientific) and stored at 4°C.

For labelling with mouse antibodies to CD63 (1B5), and PMEL (HMB45 from Dako), primary antibody was followed by rabbit anti-mouse bridging antibody (Dako). Sections were then labelled using protein-A gold as described 37.

### Immuno-fluorescence

Cells were stimulated for 25 min with 200 ng/mL Alexa647-conjugated EGF (Invitrogen) in serum-free medium prior to fixation in 4% paraformaldehyde. Cells were then stained with 100 µg/mL Filipin (Sigma) in PBS and imaged on a Leica DM-IRE2 microscope and TCS SP2 AOBS confocal system.

## Supporting Information

Figure S1 **Individual MVBs contain a range of ILV sizes.** HeLa cells were incubated with or without EGF for 25 min at 37°C and MVBs subdivided according to the presence of exclusively small (<40 nm) or large (>40 nm) ILVs or both. Most MVBs contain both small and large ILVs in resting cells but some contain exclusively small ILVs and this population is lost after EGF stimulation. EGF stimulation also gives rise to increased numbers of MVBs containing exclusively large ILVs (A). Individual ILV diameters for individual representative MVBs in resting and EGF-stimulated cells are shown in (B).

Figure S2 **High-pressure freezing confirms that MVBs in Hrs-depleted cells contain ILVs with reduced diameters.** Quantitation of high-pressure frozen cells, representative images of which are shown in Figure 6. This data shows that ILV sizes are similar in high-pressure frozen specimens to those of chemically fixed preparations and so the ‘small’ ILVs are not an artefact of chemical fixation.

Figure S3 **Accumulation of LDL-derived cholesterol in Hrs-depleted cells.** Confirming as previously shown by Du et al. (38) that Hrs depletion causes accumulation of cholesterol in endosomes. This raised the possibility that the ‘small’ ILVs might arise because of accumulation of LDL-derived cholesterol. This possibility was excluded in Figure 7.

Figure S4 **Small ILVs are generated in resting conditions and their formation is dependent on CD63.** The number of small (<40 nm) or large (>40 nm) ILVs in unstimulated control, Hrs-depleted or CD63-depleted cells were analysed. Small ILVs do form in resting conditions and their number is decreased following CD63 depletion.

Figure S5 **Simultaneous depletion of Hrs and CD63 prevents the formation of small ILVs.** The effects of depleting Hrs, CD63 or both were again analysed, using separate oligos to those used in Figure 9. The oligos used were: 5′-AAC GTC TTT CCA GAA TTC AAA-3′ (Hrs) (23), and 5′-GGA TGC AGG CAG ATT TTA ATT-3′ (CD63) (39).
