# Supplementary material for: Hrs- and CD63-Dependent Competing Mechanisms Make Different Sized Endosomal Intraluminal Vesicles
Source: Traffic. 2014 Jan 8;15(2):197–211. doi: 10.1111/tra.12139 (PMC4253088; doi:10.1111/tra.12139)

Supplementary Figure 1

A

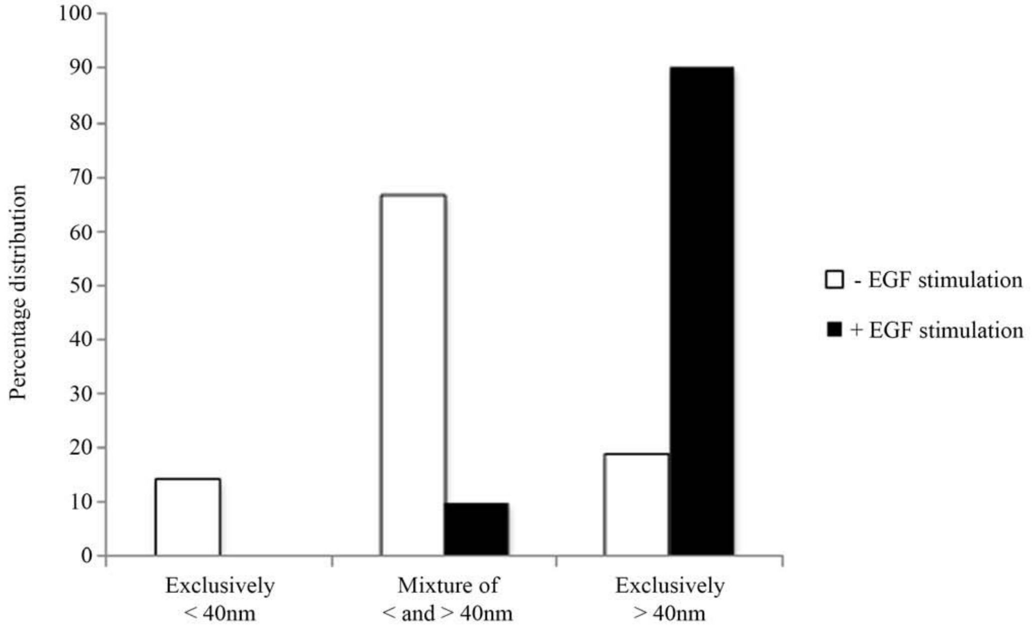

B

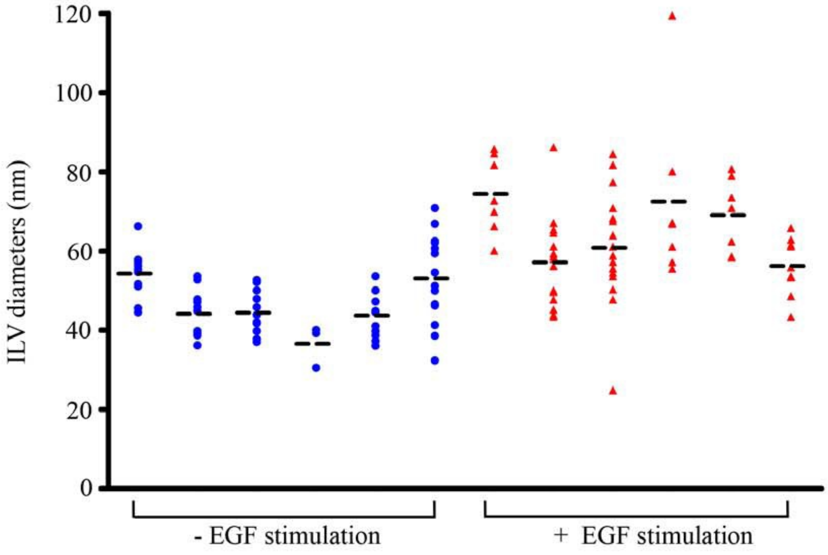

Supplement: Supplementary file 1 [file tra0015-0197-SD1.doc]
